# Percutaneous Cholecystostomy to Manage a Hot Gallbladder: A Single Center Experience

**DOI:** 10.7759/cureus.45348

**Published:** 2023-09-16

**Authors:** Mohit Bhatia, Bindhiya Thomas, Elia Azir, Doaa Al-Maliki, Khalid Ballal, Priyan Tantrige, Gibran Timothy Yusuf, Shamsi El-Hasanii

**Affiliations:** 1 Surgery, Princess Royal University Hospital, King's College Hospital NHS Foundation Trust, London, GBR; 2 Intervention Radiology, Princess Royal University Hospital, King's College Hospital NHS Foundation Trust, London, GBR; 3 Upper Gastrointestinal Surgery, Princess Royal University Hospital, King's College Hospital NHS Foundation Trust, London, GBR

**Keywords:** percutaneous biliary drainage, minimally invasive interventional radiology, conventional laparoscopic cholecystectomy, acute calculus cholecystitis, percutaneous cholecystostomy tube

## Abstract

Objective

A percutaneous cholecystostomy (PC) is a suitable option for treating acutely inflamed gallbladders. Its use has been postulated before for treating acute cholecystitis (AC), especially in elderly populations. The primary aim of our study is to analyze and present the positive results of PC as a bridge to laparoscopic cholecystectomy.

Methods

All patients who underwent PC at our hospital, Princess Royal University Hospital, King's College Hospital NHS Foundation Trust, London, GBR, from October 2020 were reviewed using a retrospective approach.

Results

Our study comprises 123 patients, with 72 females (58.5%) and 51 males (41.4%). In our study, many patients had significant comorbidities, and some of them were categorized as high-risk due to their frailty and medical conditions. The majority of the patients were in American Society of Anaesthesiologists' (ASA) groups II and III (45, 61), respectively. Though hospital stays can depend on variable factors, in our experience, the mean hospital length of stay was 12.7 days.

In our study, 119 patients (96.8%) had the procedure through the interventional radiological approach, while only four patients had it through the laparoscopic approach. The transhepatic route for drainage was more commonly practiced at our center and was used in 108 patients. At the time of writing this article, 54 patients have already had a laparoscopic cholecystectomy (LC) done as an interval procedure after surpassing the acute attack of cholecystitis, while 42 patients are still awaiting their surgical procedure.

Conclusion

Our results show that PC is a viable option, especially in cases of AC that are not responding to conservative treatments. Our study has shown low complications and conversion rates after PC. We believe PC is a safe and effective tool for managing severe and refractory cases of AC.

## Introduction

Acute cholecystitis (AC) is one of the most common emergency surgical presentations. It is estimated that around 20% of patients with gallstone disease will develop AC [1]. With the upsurge in the development of laparoscopic skills worldwide, laparoscopic cholecystectomy (LC) has been considered the mainstay for treating cholelithiasis. Laparoscopic cholecystectomy is associated with good outcomes and low morbidity; however, its role in treating acutely inflamed gallbladders, especially in older age groups, is debated due to the higher morbidity and mortality associated with it [2].

Surgical and percutaneous cholecystostomies (PCs) have been used to successfully treat patients with significant comorbidities or frailty who present with AC. In 1867, Bobbs performed the first cholecystostomy, while the first cholecystectomy was performed by Langenbuch in 1874 [3]. Overall, LC has been accepted worldwide for AC, as in acutely inflamed gallbladders, there is an increase in the conversion rates from laparoscopic to open cholecystectomy (OC), which further increases morbidity and mortality [4]. Conversion to OC is estimated to be required in up to 25% of patients with acute calculous cholecystitis. Laparoscopic cholecystectomy in the acute setting demands certain skills and has a higher risk of complications, including common bile duct injury [5]. Percutaneous cholecystostomy is believed to be a safer option for high-risk patients or patients with sepsis. It can be considered a definitive or bridging procedure for getting the patient to have an interval cholecystectomy later [6].

This study aims to display our single-center observation and approach to using PC as a bridge treatment for patients with AC until their definitive elective LC with minimal associated complications at a district general hospital.

## Materials and methods

Exclusion criteria

Patients with significant coagulation disorders, ascites, or difficulty visualizing the gallbladder were excluded. All patients who underwent PC at our hospital from October 2020 were reviewed using a retrospective and prospective approach. The data included patients’ demographics, comorbidities, length of hospital stay, time period of cholecystectomy post-cholecystostomy, and treatment outcomes. Additionally, we will discuss our views and observations on managing this common surgical emergency.

The National Institute for Health and Care Excellence (NICE) guidance suggests performing urgent LC on patients who present with AC within the first seven days [7]. According to the Association of Upper Gastrointestinal Surgeons (AUGIS) guidelines, AC should be operated on within the first three days of the onset of symptoms [8].

Practically speaking, the maximum time before attempting urgent LC will be 72 hours from the onset of the attack. Performing cholecystectomy after that will be challenging due to oedema that makes the anatomy difficult; consequently, the operation may be abandoned, ending with a partial cholecystectomy or the insertion of a surgical cholecystostomy drain. The risk of complications is high in this clinical scenario.

If patients develop symptoms of AC and present to surgical care less than three days from the onset of symptoms with imaging that confirms AC, a normal liver function test, normal amylase, and no biliary tree dilatation, then LC will be the ideal solution, provided the necessary expertise is available.

However, the practicality of everyday life in the NHS is different. Patients usually present after three days from the onset of symptoms, as they may start by self-medicating, visiting their general practitioner, or going to the emergency department. Patients tend to seek surgical help only when their symptoms are deteriorating or not resolving.

Patients who present to our unit with clinical, radiological, and biochemical features of AC beyond three days of the onset of symptoms are already treated with antibiotics and supportive treatment. If in the first 48 hours of conservative management, there is no significant response to the treatment, we proceed with percutaneous radiological cholecystostomy.

It is our clinical observation that this approach is practical and safe. It reduces the period of suffering for patients, as they usually find a dramatic improvement in their symptoms following gallbladder drainage. The length of hospital stays and the number of antibiotic doses required will be reduced when the gallbladder is drained. We usually leave the drain in situ for eight weeks before performing an elective cholecystectomy and removing the cholecystostomy drain. We found that the operation after this interval of drainage is much easier than it looks in the acute phase.

The process of thinking is changing. Previously, radiologically inserted cholecystostomy drains were offered only to patients who were very sick and unfit for surgery. This is still applicable, but our current practice is to drain the gallbladder as a bridging manoeuvre to allow the inflammation to settle before proceeding with interval cholecystectomy in eight weeks. This approach in our practice certainly avoids the possible complications and poor surgical outcomes that may occur as a result of attempting LC for acute gallbladder, especially outside the three days following an attack.

All the data were entered into a Microsoft Excel spreadsheet (Microsoft Corporation, Redmond, WA, USA) and analyzed with IBM SPSS software (IBM Corporation, Armonk, NY, USA).

Technique

All patients were assessed by the referring clinical team and showed clinical and radiological evidence of AC with failure of conservative management. Written informed consent was obtained where possible; if not possible, a decision was taken in the patient's best interests.

All the procedures were carried out by expert interventional radiologists. An initial ultrasound was performed to assess the optimal approach. A transhepatic approach was preferred, with transperitoneal use if no suitable transhepatic window was available or at the operator's discretion. If needed, a contrast-enhanced ultrasound was performed. Under sterile conditions, 1% lignocaine was administered under direct ultrasound guidance to the superficial tissue. The Seldinger technique was used under direct ultrasound guidance with a Kellet needle (Cook Medical, Limerick, Ireland) into the gallbladder. Subsequently, a stiff J-tip wire was placed into the gallbladder until resistance was felt, over which an 8 French locking pigtail drain was sited. The drain was confirmed within the gallbladder on ultrasound, and aspiration to dryness was performed. The drain was then allowed to drain freely. Analgesia and sedation were administered as required intraprocedurally with opiates and benzodiazepines. Immediate recovery was performed within the imaging department, with subsequent ward transfer. Continuous hemodynamic monitoring is performed throughout the procedure and immediate recovery. Procedural details, including complications, were recorded on handwritten notes and electronic patient records.

Contraindications included patient unwillingness, non-compliant patient, inability to visualize the gallbladder (including obstructing viscera other than the liver, e.g., colon), and significant ascites that were not resolvable with aspiration.

## Results

Demographics

**Figure 1:**
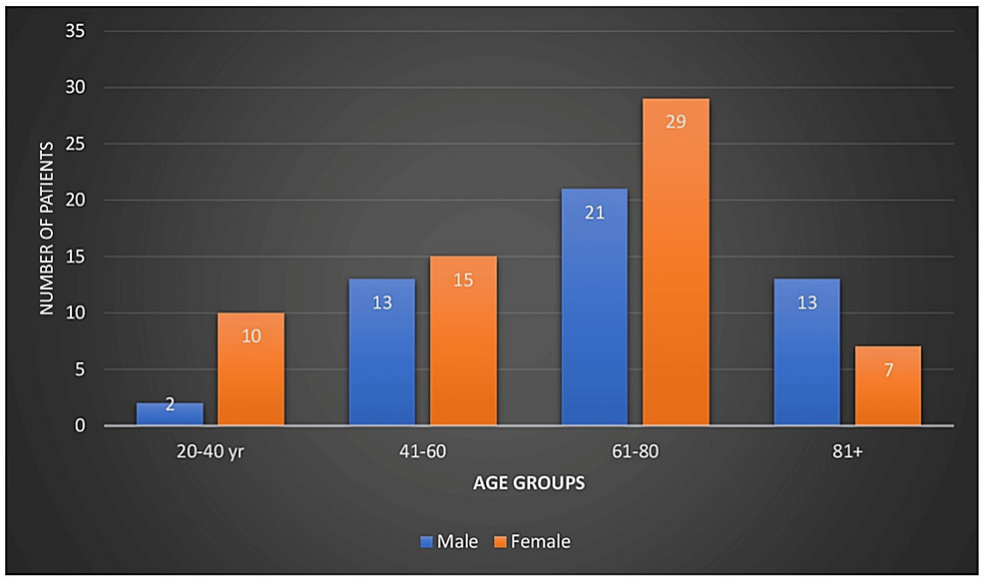
The data of the study participants categorized on the basis of age and gender

Our study comprises 123 patients, with 72 females (58.5%) and 51 males (41.4%). The majority of patients (53) were from the age group of 60-80 years (43%), followed by 30 patients (24.3%) who were older than 80 years. The demographic aspects of our study have been depicted in Table 1 and Figure 1.

**Table 1:** Age-wise distribution of the study participants

Age group (in years)	Number
20- 40	12
40 – 60	28
60- 80	53
>80	30
Total	123

Indications

**Figure 2:**
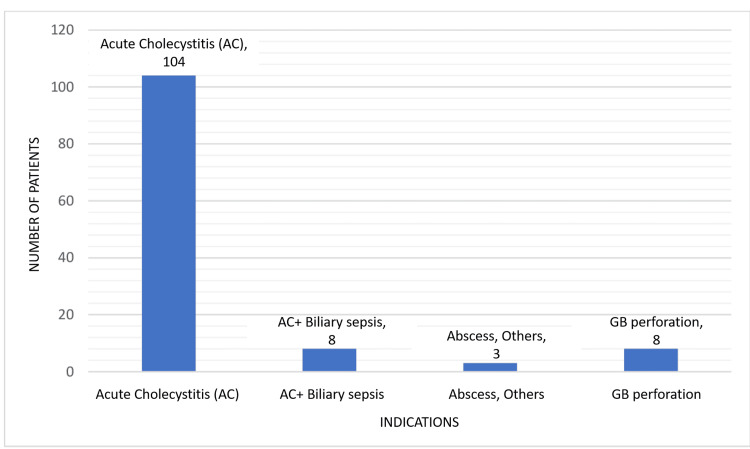
Indications for cholecystostomy AC: acute cholecystitis; GB: gallbladder

One hundred and four patients (84.6%) had episodes of AC, followed by biliary sepsis and impending gallbladder perforation. (Table 2, Figure 2)

**Table 2:** Indications for cholecystostomy in our study

Indication	Number
Cholecystitis	104
Cholecystitis + Biliary sepsis	8
Others (liver abscess)	3
Impending gallbladder perforation	8

Hospital stay

Though the length of stay in the hospital can always be variable, in our experience, the mean hospital stay duration was 12.7 days before patients were considered medically fit for discharge. The minimum and maximum durations were four and 27 days, respectively (Table 3).

**Table 3:** Length of stay in the hospital

Hospital length of stay	No. of days
Maximum	27
Minimum	4
Mean	12.1

Comorbidities

**Figure 3:**
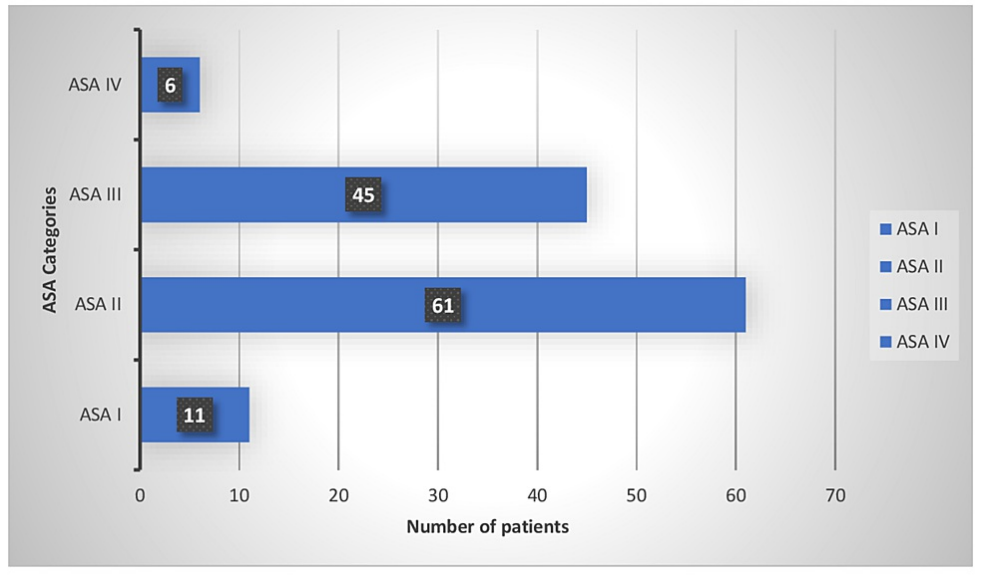
The participant data categorized on the basis of ASA levels ASA: American Society Of Anesthesiologists

In our study, many patients had significant comorbidities, and some of them were categorised as high-risk due to their frailty and medical conditions. The majority of the patients were in American Society of Anaesthesiologists' (ASA) groups II and III (45, 61), respectively (Figure 3).

In our experience, chronic pulmonary disease was the most observed comorbidity in 45 patients (36.6%), followed by other chronic and significant medical issues (Table 4).

**Table 4:** Categorization of patients based on their comorbidities

Comorbidities	No. of Patients
Chronic heart disease	38
Chronic pulmonary disease	45
Diabetes	36
Hypertension	29
Chronic smokers (current smokers?)	52

Approach

One hundred and nineteen patients (96.8%) had the procedure through the interventional radiological approach, while only four patients had it through the laparoscopic approach. The transhepatic route for drainage was more commonly practised at our centre and was used in 108 patients (Table 5, 6).

**Table 5:** Type of cholecystostomy IR: intervention radiology

Method of cholecystostomy	Number
IR-guided	119
Laparoscopic	04

**Table 6:** Approach for cholecystostomy

Method	Number
Transhepatic	108
Transperitoneal	15

In the transhepatic group, 21 out of 108 patients had recurrent cholecystitis. Whereas in the smaller transperitoneal group, only three patients had recurrent cholecystitis.

Outcomes

**Figure 4:**
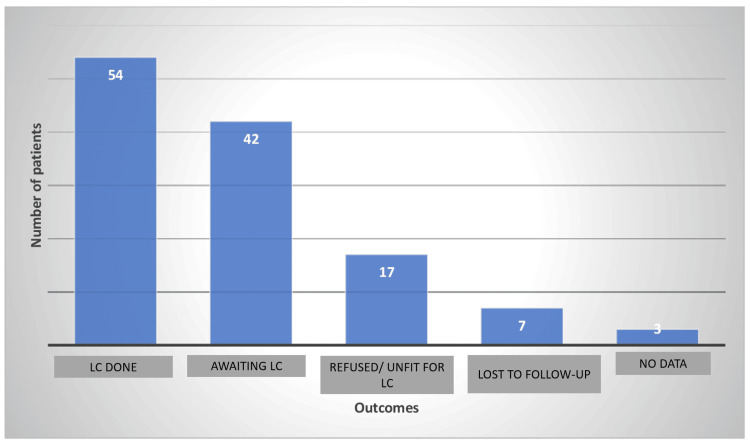
Our data after cholecystostomy LC: laparoscopic cholecystectomy

At the time of writing this article, 54 patients have already had LC done as an interval procedure after surpassing the acute attack of cholecystitis, while 42 patients are still awaiting their surgical procedure. Seventeen patients had either refused or been deemed unfit for cholecystectomy. Unfortunately, we lost seven patients to follow up with (Table 7, Figure 4).

**Table 7:** Parameters after cholecystostomy LC: laparoscopic cholecystectomy; IR: interventional radiology; PC: percutaneous cholecystostomy

Title	Number
No. of patients who had LC post IR cholecystostomy	54
No. of patients refused / unfit for LC	17
No. of patients lost to follow-up	7
No. of patients awaiting LC	42
No. available data	3
Average time period between PC and LC	127 days

Three patients aged 96, 92, and 86 had numerous comorbidities and presented with severe biliary sepsis. They were not fit for any surgical intervention, and despite PC and intensive support, they unfortunately died of sepsis. We do not feel that these three mortalities are directly related to the procedure of PC, as all these three patients are above the age of 85 with several comorbidities and presented with severe biliary sepsis, and they were not fit for any form of surgical intervention. In our series during hospitalisation, investigations revealed incidental significant malignancies which were diagnosed in four patients (two with colon cancer, one with prostate cancer, and one with breast cancer). Three patients had a bile leak early after the radiological insertion of a cholecystostomy drain and needed urgent laparoscopic intervention. One patient was managed by LC after the removal of the displaced drain.

The second patient had the drain removed, and a 22F Foley catheter was inserted as a surgical cholecystostomy. The third patient developed a bile leak at the site of the gallbladder puncture. The patient did not tolerate the procedure, and a drain could not be inserted, so laparoscopic insertion of a cholecystostomy was accomplished. All bile leaks were detected with the transperitoneal approach.

One patient was found to have drain migration during elective laparoscopic cholecystectomy. The cholecystostomy tube had eroded the colon and jejunum; the tube was removed electively with the gallbladder, and the organs were repaired. We believe this migration of the tube has to do with the severity of the initial attack of cholecystitis.

Eleven patients had their cholecystostomy tube dislodged several weeks after insertion; two of them needed reinsertion percutaneously; the other nine were asymptomatic, and the tube was removed at the bedside. Granulation tissue at the drain site and skin irritation are common problems with this treatment approach. Skin irritation and drain site granulation tissue happen in almost all patients to varying degrees.

## Discussion

Acute cholecystitis is one of the most common surgical emergencies and imposes a heavy burden on the healthcare system. It is estimated that 86% of patients with AC are treated conservatively by antibiotics or early cholecystectomy, whereas in refractory cases or high-risk patients, doing a PC is the more reasonable option [9].

The incidence of AC increases with age. Percutaneous cholecystostomy can be a life-saving procedure to treat acute sepsis, particularly in frail elderly patients with high comorbidities. Moreover, planning for interval cholecystectomy at a later stage has shown better results with low mortality rates [10]. A meta-analysis suggested that early intervention with cholecystectomy in AC is the best early treatment option [11]. However, in a country like the United Kingdom, where the mainstay of the healthcare system is public-domain hospitals, which are completely overwhelmed, providing LC for every AC patient is not feasible, even if patients present early in their attack of AC.

Within the UK, only 11% of consultants are estimated to practice early LC for acute patients. Even in the hands of highly experienced surgeons, early LC in acutely inflamed gallbladders is associated with high conversion rates, leading to higher morbidity and mortality [12]. In our study, we highlight the importance of performing PC, which has helped decrease the burden of AC on the healthcare system and provided a safe emergency pathway towards planned future surgery.

According to Yun et al. [13], 34 out of 44 patients studied underwent PC initially and subsequently had interval LC, while the rest had control of sepsis as a final procedure with PC. In our study, 112 patients had PC due to refractory AC; 54 of these patients underwent LC, with an average of 116 days passing between PC and LC. We have one patient who needs conversion to open cholecystectomy even after interval drainage of the gallbladder.

Rates of recurrent attacks of AC post-PC can vary from 6% to 40%. [14] In our study, 24 patients (22.1%) had recurrent attacks of AC post-PC. Similar results were shown by Sanjay et al., with a recurrence rate of 23% [15]. Our data show lower mortality rates, even with complicated AC cases, than some of the previous studies’ post-PC mortality rates [16]. There has been a debate on the time period for keeping the cholecystostomy drain in; many authors in the past have advocated a minimum of a six-week period for the inflammation to settle down [15]. Hsieh et al. postulated that cholecystitis recurrence increased when the drain was left in for more than two weeks [17]. In our study, we found contradicting results; the tube, on average, needed eight weeks to give us satisfactory results.

Our study suggests that a transhepatic approach for placing the cholecystostomy tube is superior to a transperitoneal approach in terms of low complication rates. This observation was also suggested by Horn et al. [18]. The selection of patients for PC has been debated in the past. Some studies have postulated its use to be limited to severely ill patients; however, some studies have suggested its application in patients with severely inflamed gallbladder with or without peri-cholecystic fluid. The presence of these factors has shown positive outcomes with PC in studies such as England et al. [19].

Many studies in the past have highlighted the importance of PC in high-risk and frail patients, presenting it as the only effective treatment for settling acute or recurrent AC attacks in that population [20]. However, there has been a rise in using PC to manage even younger patients with severe attacks of AC, and its use is gaining more acceptance [21]. Additionally, a large American group study also concluded that PC provided superior results in managing severe attacks of AC in comorbid patients. Their study showed better outcomes with PC than LC converted to OC and was found to be cost-effective as well [22]. No definitive criteria have been outlined in previous studies for selecting patients for PC [23].

Summary of our pathway

**Figure 5:**
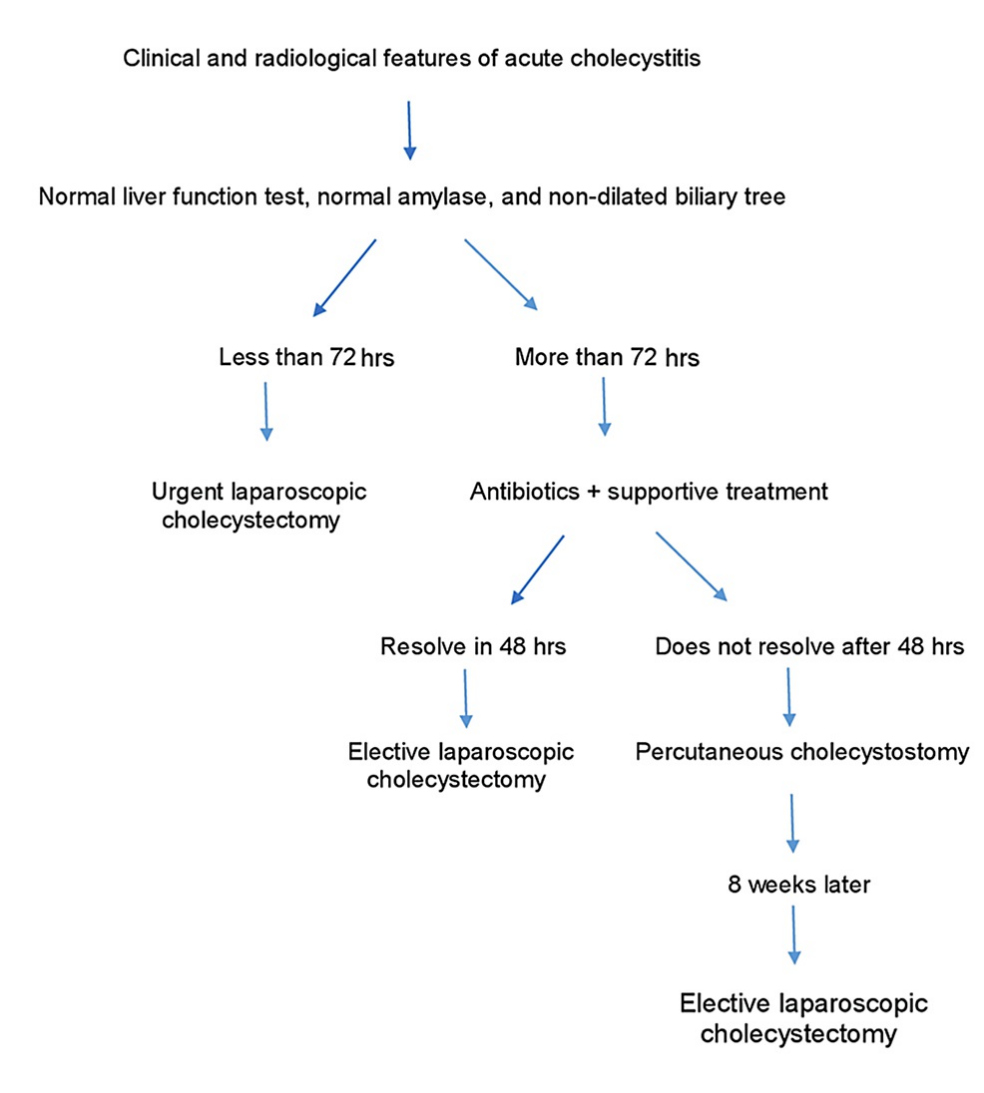
Summary of our pathway

Acute cholecystitis presented within 72 hours was radiologically proven with a normal liver function test, normal amylase, and no dilatation of the biliary tree. We proceeded to LC if feasible. Acute cholecystitis presenting after 72 hours is treated conservatively, followed by elective interval LC. Acute cholecystitis presenting after 72 hours with no clinical response to conservative management after 48 hours with distended gallbladders should proceed to PC. We leave the PC drain for at least eight weeks and remove it at the time of elective LC. Figure 5 summarizes our treatment pathway.

Limitations of the study

We have a relatively small sample size from a single centre, and the non-considered factors include postoperative time (for patients who underwent LC), quality of life with the cholecystostomy tube, postoperative analgesia requirement, and cost burden on the healthcare system. In the future, we need studies with large sample sizes that can include various parameters and give conclusive results that would benefit our fraternity.

## Conclusions

In summary, our results show that PC is a viable option, especially in cases of AC that are not responding to conservative treatments. Our study has shown low complications and conversion rates after PC. We believe PC is a safe and effective tool for managing severe and refractory cases of AC. It was our initial observation that this approach decreased the patient’s period of illness and hospital stay. This approach can also prevent complications that can result from urgent LC.
